# Single-Setting 3D MRI/US-Guided Frozen Sectioning and Cryoablation of the Index Lesion: Mid-Term Oncologic and Functional Outcomes from a Pilot Study

**DOI:** 10.3390/jpm13060978

**Published:** 2023-06-10

**Authors:** Leonardo Misuraca, Franco Lugnani, Aldo Brassetti, Loris Cacciatore, Francesco Tedesco, Umberto Anceschi, Alfredo Maria Bove, Simone D’Annunzio, Mariaconsiglia Ferriero, Salvatore Guaglianone, Riccardo Mastroianni, Gabriele Tuderti, Valeria Panebianco, Steno Sentinelli, Giuseppe Simone

**Affiliations:** 1 Department of Urology, IRCCS Regina Elena National Cancer Institute, 00128 Rome, Italy; aldo.brassetti@ifo.it (A.B.); umberto.anceschi@ifo.it (U.A.); alfredo.bove@ifo.it (A.M.B.); simone.dannunzio@ifo.it (S.D.); mariaconsiglia.ferriero@ifo.it (M.F.); salvatore.guaglianone@ifo.it (S.G.); riccardo.mastroianni@ifo.it (R.M.); gabriele.tuderti@ifo.it (G.T.); giuseppe.simone@ifo.it (G.S.); 2 Department of Urology, Hippocrates D.O.O, 6215 Divaca, Slovenia; info@lugnani.com; 3 Department of Urology, Fondazione Policlinico Universitario Campus Bio-Medico, 00128 Rome, Italy; loris.cacciatore@unicampus.it (L.C.); francesco.tedesco@unicampus.it (F.T.); 4 Department of Radiology, La Sapienza University, 00185 Rome, Italy; valeria.panebianco@uniroma.it; 5 Department of Pathology, IRCCS “Regina Elena” National Cancer Institute, 00128 Rome, Italy; steno.sentinelli@ifo.it

**Keywords:** prostate cancer, focal therapy, frozen section, index lesion, MRI-guided biopsy, cryoablation, primary cryoablation treatment, cryotherapy

## Abstract

Our study explored frozen section reliability in prostate cancer (PCa) diagnoses and described surgical steps of a 3D magnetic resonance imaging (MRI)–ultrasound (US)-guided prostate biopsy (PB) and focal cryoablation of the index lesion (IL) in a single-setting procedure. Patients with a suspicious prostatic specific antigen (PSA) value, with a PIRADS 4 or 5 single lesion, were enrolled for trans perineal 3D MRI–US-guided PB and TRUS-guided focal cryoablation. Three cores were taken from the IL, three cores from the surrounding area, while systematic sampling was performed for the rest of the gland. After confirmation of PCa in frozen sections, focal cryoablation was performed. The 1st-year follow-up schedule included a PSA test at a 3-month interval, MRI 3 months and 1 year postoperatively and PB of the treated area at 1 year. Following the follow-up schedule, an involved PSA test at a 3-month interval and yearly MRI were performed. The PCa diagnosis was histologically confirmed in all three patients with frozen sections. At final histology, a single Gleason score upgrade from 6 (3 + 3) to 7 (3 + 4) was observed. All patients were discharged on postoperative day 1. At the 3-month evaluation, mean PSA values decreased from 12.54 (baseline) to 1.73 ng/mL and MRI images showed complete ablation of the IL in all patients. Urinary continence and potency were preserved in all patients. At the 1-year follow-up, one patient had suspicious ipsilateral recurrence on MRI and underwent a new analogous procedure. Post follow-up was uneventful and PSA remained stable in all patients. Three-dimensional MRI–US-guided frozen sectioning and focal cryoablation of the IL is a step forward towards a “patient-tailored” minimally invasive approach to the diagnosis and cure of PCa.

## 1. Introduction

The rise of novel and innovative technologies in the surgical armamentarium represents a main interest in the urological scenario. Among the various niches of urology, prostate cancer (PCa) depicts the utmost representation of these ancillary minimally invasive techniques. PCa is the second most common cancer in men worldwide [1], with a different prevalence depending on geographical areas and ethnicity [2]. Additionally, the incidence of a PCa diagnosis varies across different geographical locations, due to the varying recommendations on screening determined by international organizations, essentially based on the prostate-specific antigen (PSA) test [1]. In the PSA era, improvements in magnetic resonance imaging (MRI) have led to the better defining of which patients could deserve a prostate biopsy and more interestingly the identification of the index lesion. Parallelly, the development of MRI–ultrasound (US) fusion imaging and the improvement in MRI-targeted biopsy techniques [3,4] have led to significantly higher accuracy in the diagnosis of PCa. Consequently, their use has been increasingly adopted to guide prostate biopsies. Indeed, MRI-guided biopsy procedures allow for displaying the needle and guiding it within the prostate parenchyma in the multiplanar mode, as opposed to an ultrasound-guided procedure, in which the insertion of the needle and subsequent biopsy sampling can be performed only in a single plane [5].

In recent years, the extremely high precision developed in prostate biopsy performing laid the foundation to the birth and diffusion of focal therapy, which has sought to bridge the existing therapeutic empty space between two diametrically opposite solutions: active surveillance on the one hand, and radical prostatectomy and radiotherapy on the other hand. Moreover, several minimally invasive procedures have been developed with the purpose to ensure the same oncological safety compared to radical surgical treatment or radiotherapy, reducing at the same time both intra- and post-operative complications, and improving functional outcomes that have less impact on urogenital function. In this setting, high-intensity focused US (HIFU), cryotherapeutic ablation (cryotherapy) and focal photodynamic therapy (PDT) of the prostate, with whole-gland, mid-gland or focal lesion treatment [6,7], have emerged as ablative techniques. In more detail, cryotherapy uses freezing cycles through the placement of cryo-needles under TRUS guidance, in order to induce cell death due to a different mechanism determined by ice crystals, such as protein denaturation, the direct rupture of cellular membranes, vascular stasis and microthrombi formation, resulting in stagnation of the microcirculation with subsequent dehydration and ischemic apoptosis [8,9,10]. In the field of cryoablation, there are several procedural differences between the use of ultrasound and MRI to guide the treatment for local lesion PCa. Specifically, ultrasound-guided cryoablation is affected by a poor quality of images with a significant drawback in monitoring the ablated zone due to creation of the iceball during the treatment, leading to the angle-shadowing effect [11]. Conversely, in MRI sequences, the solid structure of the iceball determines ultra-short T1 and T2 relaxation times, enhancing the contrast with the adjacent soft tissues [12]. Furthermore, thanks to multiplanar imaging, the extension and the propagation of the iceball can be more accurately monitored in order to avoid its contact or diffusion with non-target adjacent structures, such as the rectal wall, the external sphincter and the neurovascular bundles [12,13].

No matter the energy or the process used to ablate prostatic tissue, to perform any focal therapy, a histopathological confirmation of the PCa diagnosis is mandatory to move from biopsy towards treatment. Therefore, nowadays, urologists are obliged to defer treatment from biopsy after pathological report availability. Our pilot study demonstrates the safety and feasibility of a unique procedure, which comprises a diagnosis and treatment in the same surgical session, removing the waiting time between prostate biopsy and focal cryoablation, creating a “patient-tailored” minimally invasive approach for the identification and cure of a PCa index lesion. We thereby describe step-by-step the 3D MRI–US-guided frozen sectioning of a prostate biopsy and subsequent focal cryoablation of the index lesion in a single setting approach in our tertiary referral–center, and we report mid-term oncologic and functional outcomes.

## 2. Materials and Methods

### 2.1. Patient Selection

In our study, we considered only patients suspected of having PCa, based on PSA value, and MRI-proven PIRADS 4 or 5 single lesions. A metastatic evaluation with total body PET or CT plus bone scan was also performed for selected patients with PSA > 10 ng/mL to exclude those with possible non-organ confined disease. After a detailed discussion about the limitations and benefits of focal cryoablation and the reliability of the frozen section for PCa, only patients who refused any radical treatment were enrolled to perform this treatment at our tertiary–referral institution.

Patients were evaluated by history, physical examination, routine preoperative urine (chemical–physical urine analysis and urine culture), blood tests (blood count, electrolytes and creatinine), PSA test, the use and number of pads for incontinence and validated questionnaires for sexual function (the International Index of Erectile Function, IIEF-5 [14]), and for lower urinary tract symptoms (LUTS) (the International Prostate Symptom Score, I-PSS [15]).

### 2.2. Preoperative Preparation

On the day before surgery, patients were given a laxative. Moreover, patients fasted for a minimum of 8 h, and 2 h before procedure, they received an enema and prophylactic antibiotic therapy intravenously (basically cefazoline 2 g or ciprofloxacin 400 mg in case of cephalosporin allergy).

### 2.3. Surgical Technique

Fusion biopsy and cryoablation were performed in the same setting, as a 1-day surgery. The entire procedure was performed transperineally, with the patient under spinal anesthesia, in lithotomic position. The perineum was previously shaved and prepared, and the scrotum was suspended while an 18-French Foley catheter was inserted. Bladder was emptied and refilled with 100–150 mL of sterile saline solution. In the meantime, the antibiotic was administered intravenously.

We used the NavigoTM (UC-CARE Medical Systems, Yokneam, Israel) and an argon/helium gas-based third generation cryoablation system (Endocare; HeathTonics Inc., Austin, TX, USA). The NavigoTM System provides a 3D real-time orientation of both probe and targeted prostate areas. This real-time adjustment is generated thanks to an electromagnetic transmitter above the body sensor and the probe sensor. This tracking system allows both transrectal and transperineal biopsies, and it can be combined with the US based on Endocare^®^ Cryocare^®^ Systems.

#### 2.3.1. MRI–US Fusion Biopsies of the Index Lesion

After a US scan of the entire gland, the prostatic diameters were calculated using the NavigoTM System, while prostatic contours were scored and marked on the screen. MRI images previously uploaded on NavigoTM System were fused with US images and a 3D model was created by NavigoTM software. The index lesion, previously marked on MRI images, was clearly integrated and visible in the 3D model. Every prostatic biopsy was performed under MRI–US guidance, using a grid to track the exact location of prostate biopsies and as a guide for subsequent cryoprobe insertion.

**Figure 1:**
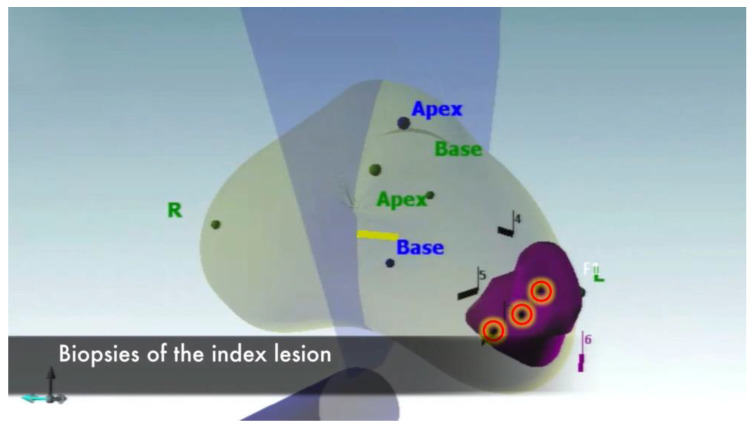
Bioptic sampling of the index lesion.

Overall, three cores were taken from the index lesion (Figure 1), one of which was sent to the frozen section. Other two cores were sent for final histopathological examination to confirm Gleason score and to provide an accurate tumor staging.

#### 2.3.2. Biopsies of the Area Surrounding the Index Lesion

**Figure 2:**
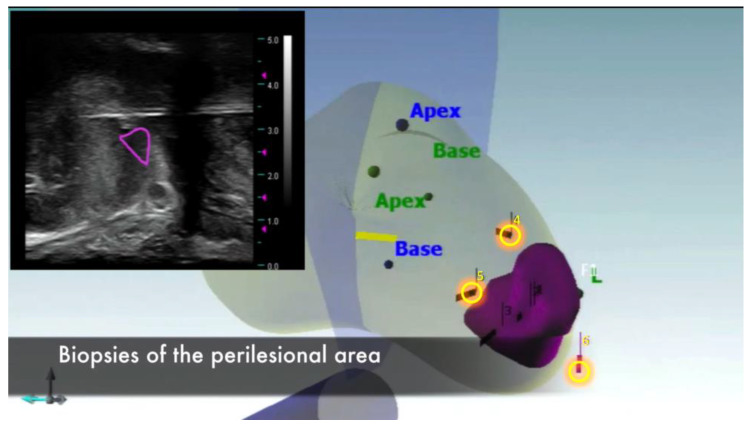
Bioptic sampling of the perilesional area.

Three cores were taken from the perilesional area (Figure 2) and they were sent to the frozen section evaluation to ensure a safety treatment margin of index lesion ablation. The frozen section of these three cores must be negative to proceed with performing focal cryoablation. In case of positive core at one of these sites, a further biopsy should be performed at a distance of 5 mm to ensure proper safety margins.

#### 2.3.3. Systematic Prostate Biopsy

**Figure 3:**
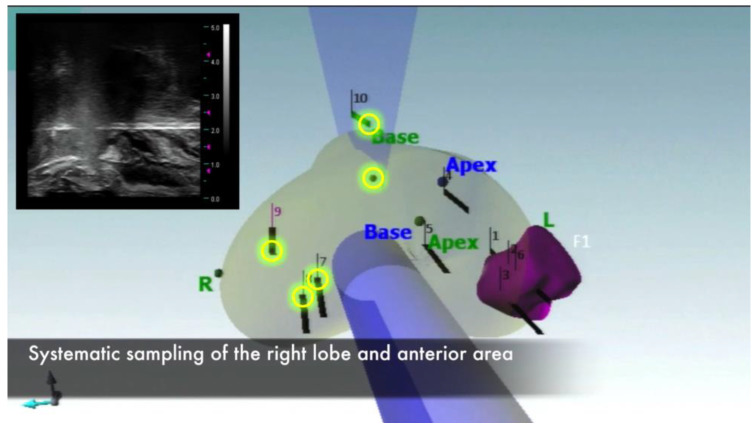
Systematic 6-core sampling of the contralateral lobe.

A systematic 6-core sampling of the contralateral lobe was performed (Figure 3) and cores taken were sent to the frozen section to exclude other PCa foci.

#### 2.3.4. Frozen Section

**Figure 4:**
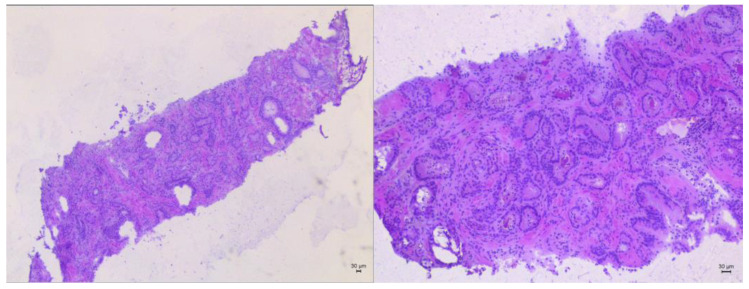
Frozen section showing prostate cancer Gleason score 6 (3 + 3) (Hematoxylin/Eosin).

Frozen sections were used for different purposes: first of all, to confirm the presence of PCa at the level of MRI index lesion (Figure 4), then to shape the treatment area of focal cryoablation, obtaining negative cores at all three perilesional cores, at a minimum distance of 5 mm from the index lesion margins. Finally, they were used to exclude the presence of clinically significant PCa [16] in the rest of the gland.

#### 2.3.5. Procedure Planning

The 3D model created by NavigoTM allows us to accurately define the area to be treated. Cryoablation planning is a crucial step to determine which probes, how many of them to use and where to position them. The aim is creating an iceball shape to completely inscribe the treatment area.

#### 2.3.6. Cryoprobe and Thermocouple Placement

**Figure 5:**
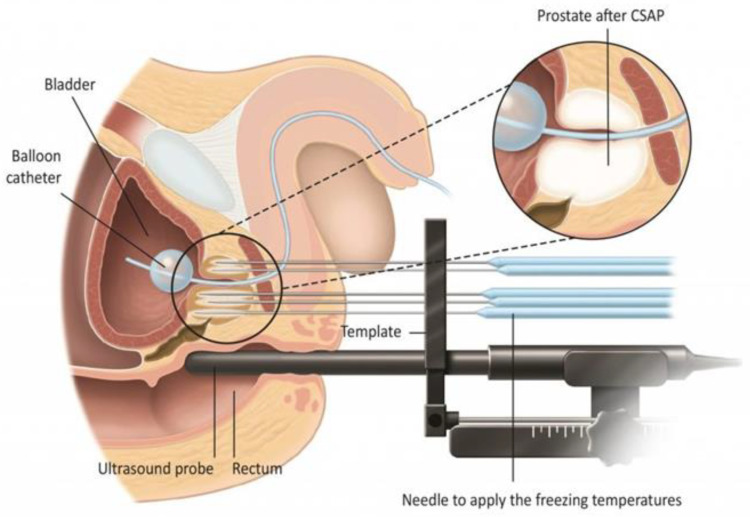
Description of cryoablation technique and arrangement of cryoprobes on “https://patients.uroweb.org (accessed on 18 May 2020)”. The European Association of Urology Patient Information (EAU PI) delivers, with the support of EAU guidelines [17], education, images and video content with a language easy to understand for patients.

**Figure 6:**
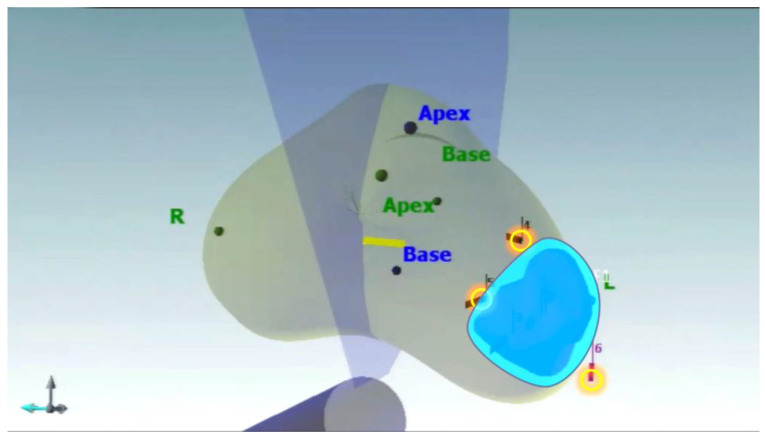
Cryoprobe placement and shaping of the iceball tailored to the planned treatment area.

Cryoprobes and thermocouples were placed transperineally under transrectal US guidance as shown in Figure 5. We basically used three to four cryoprobes that were placed around the index lesion, exactly at the level of the perilesional biopsies. We specifically used V-ProbeTM by EndoCare, which can create variable iceball sizes from 1.5 to 5 cm by sliding a blue tab to the desired isotherm length. It is perfectly suited to shape an iceball tailored to the planned treatment area (Figure 6). This permits us to minimize any unintended injury to surrounding tissues while precisely targeting the index lesion. A thermometer probe was inserted into the index lesion, where the frozen section had confirmed the presence of PCa to allow active monitoring of the target temperature during the entire freezing process. A second thermometer probe was placed at the level of the external sphincter to guarantee the maintenance of a temperature greater than 0 °C during the entire treatment. An additional third thermocouple can be positioned near the neurovascular bundle to ensure the maintenance of the correct temperature to avoid any unintended injury at this level.

#### 2.3.7. Urethral Warming Catheter Placement

Urethral warming catheter was placed before starting cryoablation. It is a dual lumen urethral catheter that can be passed transurethrally to circulate warm saline and prevent the destruction of the urethral epithelium. This prevents transmural necrosis, maintaining the epithelial barrier for the containment of necrotic prostate tissue after treatment [18].

Setting the temperature of saline solution, which circulates into the dual lumen, permits establishing the thickness of preserved urethral tissue. In case of tumoral foci near the urethra, a reduced temperature can guarantee a more complete ablation, reducing the risk of undertreatment and residual disease.

At the end of the procedure, urethral warming catheter was replaced with a Foley catheter, which was removed on the 7th postoperative day.

#### 2.3.8. Focal Cryoablation

Focal cryoablation was performed with two freezing cycles as standard. A homogeneous temperature reduction was observed on the screen while the US imaging showed the progressive formation of the iceball. The target temperature of −40 °C was maintained for at least 2 min per freezing cycle, for a total time of more than 4 min. A passive thawing phase of 15 min was ensured between the two consecutive freezing cycles.

### 2.4. Postoperative Course and Follow-Up

**Figure 7:**
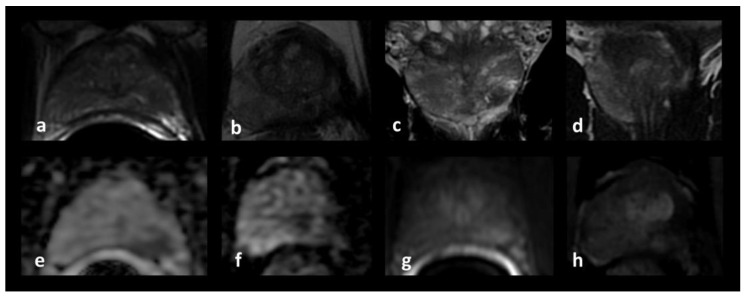
Pre-operative and post-operative multiparametric MRI images. (**a**): Pre-operative axial T2-weighted image. (**b**): Post-operative axial T2-weighted image. (**c**): Pre-operative coronal T2-weighted image. (**d**): Post-operative coronal T2-weighted image. (**e**): Pre-operative axial ADC (apparent diffusion coefficient) map image. (**f**): Post-operative axial ADC map image. (**g**): Pre-operative axial perfusion image. (**h**): Post-operative axial perfusion image.

Patients were discharged on the 1st postoperative day. Foley urethral catheter was removed on the 7th postoperative day. Continence was defined as the absence of any urine leakage. Erectile function was assessed by means of IIEF-5 score. LUTS were assessed using the I-PSS scoring system [15]. PSA test was evaluated 1 month after surgery and every 3 months subsequently for the 1st year. Three-month (Figure 7) and one-year MRI was performed [19,20,21] to confirm complete ablation of the treated area. Following follow-up schedule, involved PSA test at a 3-month interval and yearly MRI were performed.

On the base of 1-year MRI, a targeted biopsy of the treated area and of all suspicious metachronous lesions was performed according to the International Multidisciplinary Consensus on Trial Design for focal therapy in PCa [22,23]. Quality of life was assessed using the Expanded Prostate Cancer Index Composite (EPIC) questionnaire (version 2.2002, 32 items) [24].

## 3. Results

Overall, three patients underwent 3D MRI–US-guided frozen sectioning and focal cryoablation of the index lesion with the described technique. All patients had a clinical suspicion of PCa based on the PSA test and a single MRI lesion with a PIRADS score of 4 or 5 and denied consent to any radical treatment. They were all potent and continent and complained of no relevant LUTS before treatment. Baseline clinical data and 3-month, 1-year, 3-year and 5-year functional and oncologic outcomes were collected and reported (Table 1, Table 2, Table 3, Table 4 and Table 5).

Erectile function and urinary continence were preserved in all patients. Median IIEF-5 decreased slightly from 19 to 18 after treatment. All patients continued to use any pad, and the median I-PSS score changed faintly from 10 to 9 after cryoablation. The postoperative course was uneventful, and all patients were discharged on the 1st postoperative day.

Quality of life, assessed using the EPIC questionnaire, showed no clinically meaningful changes after treatment. The PCa diagnosis was histologically confirmed in all cases with frozen sections, which revealed a Gleason score of 6 (3 + 3) in all three patients. At final histology, there was a Gleason score upgrade in one patient from 3 + 3 to 3 + 4. Mean PSA values decreased from 12.54 (baseline) to 1.73 ng/mL at the 3-month evaluation, to 2.31 ng/mL at the 1-year follow-up and to 2.71 ng/mL at the 5-year follow-up. Mean PSA reduction was 86.9%, 82.3% and 78.4% at the 3-month, 1-year and 5-year evaluation, respectively. Three-month postoperative MRI images showed complete ablation of the index lesion in all patients. At the 1-year follow-up, neither MRI imaging nor treated-area biopsies showed signs of persistent or recurrent disease in two out of three patients.

In one patient, a suspicious area was observed at 1-year MRI and retreatment was performed; the final histology confirmed a 4 + 3 ipsilateral PCa recurrence previously identified on the frozen section. At the following MRI performed yearly, no new onset PIRADS ≥ 3 lesions were identified in all patients. At the 3-year and 5-year evaluation, two patients showed mild LUTS, while one patient had moderate symptoms, and two patients showed mild to moderate and one patient showed mild erectile dysfunction. At the 5-year follow-up, all patient-maintained PSA values were comparable to PSA nadir after treatment and no new onset PIRADS ≥ 3 lesions were identified.

## 4. Discussion

In the MRI era, the accurate detection of clinically significant prostate cancer is still challenging for urologists worldwide. In this scenario, it has been shown how MRI is able to detect significant PCa in biopsy-naïve males with prior negative biopsies. Indeed, according to a systematic review of the literature, the negative predictive value of mpMRI ranges from 63 to 98% [25]. This indicates that in the presence of an index lesion without further foci, the risk of missing PCa is poor and consequently the risk of missing clinically significant cancer is reduced up to 2–7%. Therefore, Fütterer et al. [25], in their systematic review, reported that MRI could be used to rule out significant disease and patients with suspicious PSA may obtain fewer samples, undergoing a targeted biopsy and avoiding systematic sampling. Despite the significant advancements in imaging technologies, the diagnosis of PCa is still based on prostate biopsy [26], which has itself potential complications with conceivable impact on quality of life [27]. In the attempt to reduce them, less numerous and more precise biopsies have been advocated. In this context, recent studies demonstrated that fusion biopsies outperform systematic biopsies, increasing the detection rate of clinically significant PCa [28,29]. Nevertheless, the treatment of PCa must be postponed at the availability of a pathologic report of prostate biopsy, exposing the patient to the possible consequences of two separate and distinct surgical procedures.

Our study is a proof of concept that demonstrates feasibility and safety of performing a prostate biopsy and focal treatment in the same surgical session. According to the IDEAL framework reported by McCulloch et al. [30], our manuscript could be considered as a stage 1 study. Even though in our small series the initial diagnosis of PCa with frozen sections was confirmed in all cases, one case revealed an upgrade in the Gleason score. Therefore, more robust evidence is awaited to confirm reliability and diagnostic accuracy of frozen sections for PCa, similarly to several oncological contexts, such as ovarian cancer [31] and endometrium [32]. This lays the foundations of a possible fusion of the diagnostic and therapeutic moment in those patients who are candidates for focal therapy with an expected reduction in costs, patients’ distress and the waiting list. In recent years, a few studies investigated the consistency of frozen sections in the urologic field and specifically regarding prostate cancer. Dinneen et al. [33] reported that systematically performed frozen sectioning of the posterolateral prostate margin can facilitate preservation of neurovascular bundles, with a consequent modest reduction in positive surgical margin (PSM) rates. However, no high-level evidence exists, and no strong recommendations can be drawn about the use of frozen sections in identification of prostate cancer foci.

Although PCa is a multifocal pathology in 60–90% of patients, it is often possible to identify a dominant lesion, called the “index lesion”, characterized by a higher Gleason score than satellite lesions. This area is likely to be the tumor area that will drive a patient’s prognosis [34]. This assumption represents the rationale behind all organ-sparing therapies that aim for adequate oncological control through the sole treatment of the dominant lesion, minimizing undesired effects related to radical therapeutic options. Recent scientific evidence suggests that the treatment of the index lesion is feasible, oncologically safe and characterized by excellent functional results [35,36,37]. Ahmed et al. [38] showed that focal therapy of the index lesion is a feasible, safe and well-tolerated treatment, with high rates of genitourinary functional preservation. A total of 58.9% of patients were leak-free, pad-free continent and had erections sufficient for penetration at the 12-month evaluation. Another two studies focused their attention on comparing the whole gland versus focal cryoablation. Bossier et al. [39] reported no difference in pad-free continence between groups (both 83%), while Mendez et al. [40] described a significant difference in favor of focal cryotherapy (24-month continence: 100% vs. 98.7%, *p* = 0.02). Moreover, the latter study also demonstrated a significant better recovery of erectile function for the focal treatment group (68.8% vs. 46.8% at the 24-month evaluation, *p* = 0.001) [40].

Despite the small sample size, the diagnostic accuracy of frozen sections appeared comparable to that obtained by means of conventional prostate biopsy. Frozen section findings were confirmed through final histopathological evaluation three out of four times (during two out of three primary cryoablations and at the unique retreatment).

Our study introduces several original messages from a clinical standpoint: first of all, we report the feasibility of combining the diagnosis and focal treatment of PCa in a single surgical session; secondly, we confirm the ablative effectiveness of PCa focal cryoablation by means of a 1-year prostate biopsy and the mid-term oncologic efficacy by means of MRI performed yearly; we report the immediate, and maintained over time, functional benefits of such treatment in terms of erectile function and urinary continence preservations and finally the negligible impact on patients’ QoL assessed with the EPIC questionnaire [24]. We also testified the feasibility of retreatment in a patient who had an ipsilateral recurrence at the 1-year follow-up and the reliability of mpMRI to assess the overtime risk of PCa recurrence after treatment.

Our study is not exempt from criticism and limitations: first of all, the small sample size, essentially due to the “pilot-study-design” and the low enrollment that basically requires patients’ motivation to refuse any kind of radical treatment; the need for trust in the diagnostic accuracy of mpMRI, with the consequent risk of omitting the diagnosis of other PCa foci with performing a 12-core “only” systematic prostate biopsy; and the need for an expert uropathologist to optimize the diagnostic accuracy of frozen sections and therefore the risk of poor reproducibility in other centers.

Finally, defining the proper indication of MRI–US-guided biopsy and focal cryoablation is beyond the scope of this study and will certainly require larger populations and reports from other centers. Similarly, a precise follow-up schedule after a focal treatment is still awaited and based on the International Multidisciplinary Consensus on Trial Design [22] with the potential risk of missing the “lethal clone” by treating the index lesion exclusively [41]. A continuously increasing role of multiparametric MRI in different contexts, including diagnoses, treatment, surveillance and follow-up, is likely to be anticipated [42,43].

## 5. Conclusions

Our pilot study highlights the reliability of frozen sections in the context of PCa and demonstrates safety and feasibility of a single surgical procedure that merges a diagnosis and treatment. Achieving the diagnosis and focal treatment of a PCa index lesion in a single session is a further step towards a minimally invasive and patient-tailored approach. An ongoing prospective trial with subsequent adequate follow-up will confirm the reliability of frozen sections for PCa diagnoses and the therapeutic effect of 3D MRI–US-guided focal cryoablation of the index lesion.

## Figures and Tables

**Table 1:** Demographic and baseline clinical data.

Characteristics	Patient 1	Patient 2	Patient 3
Age, y	49	79	76
PSA, ng/mL	11.69	11.42	14.51
Body mass index, kg/m^2^	26.7	24.6	28.3
Digital rectal examination	Negative	Negative	Negative
MRI findings	PIRADS-5	PIRADS-4	PIRADS-4
cT stage	cT3a	cT2b	cT2a
ASA score	1	2	2
Index lesion volume, cc	17	25	16
IIEF-5 score	19	16	21
I-PSS score	4	10	12

**Table 2:** Three-month postoperative data.

Characteristics	Patient 1	Patient 2	Patient 3
PSA, ng/mL	0.8	1.23	3.15
Digital rectal examination	Negative	Negative	Negative
MRI findings	Negative	Negative	Negative
Continence, yes/no	Yes	Yes	Yes
IIEF-5 score	21	16	18
IPSS score	5	12	9

**Table 3:** One-year postoperative data.

Characteristics	Patient 1	Patient 2	Patient 3
PSA, ng/mL	1.63	1.34	3.96
Digital rectal examination	Negative	Negative	Negative
MRI findings	Negative	Negative	PIRADS-4
Repeated biopsy	Negative	Negative	4 + 3
Continence, yes/no	Yes	Yes	Yes
IIEF-5 score	20	18	16
IPSS score	4	11	10

**Table 4:** Three-year postoperative data.

Characteristics	Patient 1	Patient 2	Patient 3
PSA, ng/mL	1.87	2.11	2.87
Digital rectal examination	Negative	Negative	Negative
MRI findings	Negative	Negative	Negative
Continence, yes/no	Yes	Yes	Yes
IIEF-5 score	19	15	15
IPSS score	7	13	9

**Table 5:** Five-year postoperative data.

Characteristics	Patient 1	Patient 2	Patient 3
PSA, ng/mL	2.51	3.16	2.47
Digital rectal examination	Negative	Negative	Negative
MRI findings	Negative	Negative	Negative
Continence, yes/no	Yes	Yes	Yes
IIEF-5 score	18	15	12
IPSS score	8	14	6

## Data Availability

The data reported in this paper are available from the corresponding author upon request. Data is not publicly available due to privacy or ethical restrictions.
